# “Empowerment” without transformation? A critique of women empowerment without a masculinities lens in gender and agriculture literature

**DOI:** 10.3389/fsoc.2026.1636863

**Published:** 2026-02-20

**Authors:** Amon Ashaba Mwiine, Margaret Najjingo Mangheni, Martha Businge, Fred Shimali, Stephen Angudubo, Brenda Nakyewa, Grace Nanyonjo, Elizabeth Asiimwe, Losira Nasirumbi Sanya

**Affiliations:** 1 Department of Women and Gender Studies, Institute of Gender and Development Studies, Makerere University, Kampala, Uganda; 2 Department of Extension and Innovation Studies, Makerere University, Kampala, Uganda; 3 Gender-responsive Researchers Equipped for Agricultural Transformation (GREAT) Project, College of Agricultural and Environmental Sciences, Makerere University, Kampala, Uganda; 4 National Crop Resources Research Institute (NACRRI), Namulonge, Uganda; 5 Department of Agriculture, Faculty of Agricultural Sciences, Uganda Christian University, Mukono, Uganda

**Keywords:** agriculture, male involvement, masculinities, transformation, women’s empowerment

## Abstract

Women empowerment in pursuit of gender equality has received much attention in agriculture and development practice literature. At the same time, there is increasing realization of the need for interventions that focus on masculinities in order to ensure sustainable transformation of social/gender relations in agricultural communities. However, a review of literature on women empowerment in agriculture over the period 2010 to 2022, targeting Sub-Saharan Africa and Asia revealed that notions of women empowerment and masculinities have been mostly studied and applied in agricultural research independent of each other and rarely in an interconnected manner. We argue that research and interventions on women empowerment without a masculinities lens pause a risk of ‘empowerment without transformation’ especially when root causes of unequal gender relations in farming communities are not challenged and/or are inadvertently reproduced.

## 1 Introduction

Discussions about women’s empowerment and notions of men and masculinities have gained traction as part of the global strategic interventions to address gender inequalities (Ambler et al., 2021; Casey et al., 2016; FAO et al., 2020; Lecoutere and Wuyts, 2021; Santoso et al., 2019). Women’s empowerment—the expansion in women’s ability to make strategic life choices (Santoso et al., 2019)—and its explicit engagement with men and boys, are interventions particularly being drawn upon in food security and broadly agricultural research to promote the transformation of norms that perpetuate gender inequality (Hillenbrand and Miruka, 2019). Unwritten, informal rules on who a farmer is, division of crops and plots/fields, decision making patterns along the agricultural value chain, agricultural assets accumulation and ownership of productive resources, social norms around land inheritance and land rights, and many other aspects often play out differently for men and women farmers. Because social gender norms have implications for men and women, transformation of gender relations in agricultural communities increasingly requires interventions that work with couples who learn together and apply the learning in all spheres of their life (FAO et al., 2020).

There is a growing body of literature pointing to how, at times, agriculture interventions designed to empower women proceed as if women are isolated from and act independent of other household members, especially the men (Ambler et al., 2021; Bonatti et al., 2019; Cole et al., 2015; FAO et al., 2020; Sharaunga et al., 2015). In some studies [see for instance, Bonatti et al. (2019)], women’s empowerment interventions often triggered tensions in households that manifested in form of men’s violence against women. In other studies such as Ambler et al. (2021), the authors acknowledge that most of the micro-interventions on empowerment tend to focus exclusively on women small holder farmers in resource-poor communities. However, they subtly acknowledge the dilemma that comes with programmatic focus on women alone yet women’s everyday lives are intricately woven with other members of society. They argue, “it is important to recognize that a married woman’s level of empowerment may depend directly on others in the household with whom she must negotiate when making decisions. As such, efforts to increase her empowerment may be more effective if others (namely her husband) are receptive” (Ambler et al., 2021, p. 2). On the one hand, these submissions subtly point to possibilities of empowerment efforts with limited outcomes on transformation of unequal gender relations as well as outright resistance to women’s empowerment and gender equality. Studying an economic intervention where male farmers were expected to transfer sugar cane blocks to their wives, the study not only reported men’s reluctance to register sugar cane blocks in their wive’s names but also a host of unintended impacts on women’s other productive activities and time use, the time use of others in the household, and the household’s livelihood diversification. On the other hand, they alert us to the possible value of collaboration, cooperation, and joint efforts between women and men towards collective conscientization, questioning, and transformation of household gender relations.

Some studies highlight the idea of “working with men” or “male involvement” in women’s empowerment and gender equality (Bonatti et al., 2019; Brandth, 2019; Lecoutere and Wuyts, 2021; Sraboni et al., 2014). For instance, Bonatti and colleagues highlight the inherent complexity of develop solutions and actions that improve the lives of those living in hunger while simultaneously addressing gender relations. They point out complex relationships between undernutrition, violence, food, and gender as much as they demonstrate inter-relations between women and men’s everyday lives. Yet, the interrogation of men as a gendered category whose lives are woven with women’s everyday experiences often remains unexamined or taken for granted (Cornwall, 2000) in many other studies. In her work, *Men missing in GAD?* Cornwall, elaborates on ways in which,

*“Men, in all their diversity, have until recently been largely missing from GAD discourse. Their occasional appearances tend to be in the guise of Man the Oppressor, as custodians and perpetrators of male domination and as obstacles to equitable development. …. Rarely if ever are men depicted as people—sons, lovers, husbands, fathers—with whom women might have shared interests and concerns, let alone love and cherish. Nor is the range of subject positions actual men may occupy in different kinds of relationships with women, or indeed men, brought into the frame. Rather, ‘men’ emerge as a potent, homogeneous category that is invariably treated as problematic”* (Cornwall, 2000, pp. 18–19).

Cornwall raises concerns that studies on women’s empowerment are increasingly acknowledging, i.e., the constitution of ‘women’ and ‘men’ as binary categories in opposition to each other. A similar discourse constitutes women’s empowerment as a ‘zero-sum’ game in which there are losers and winners, and consequently the constitution of men in homogenizing ways as perpetrators of male domination and obstacles to women’s progress.

Despite the increasing turn towards designing agricultural development interventions working on women’s empowerment to *include* men’s participation, there remains a significant body of knowledge that conceptualizes women’s empowerment in agriculture without a masculinities lens. The paper therefore looks at how these concepts are defined and applied in agriculture literature and why there is an increasing push to focus on these notions in an interconnected manner. We specifically address the questions: *How does the literature conceptualise women empowerment and masculinities? How does it explain the links between concepts of women empowerment and masculinities?* We reflect on how research and interventions on women’s empowerment without a masculinities lens might lead to ‘empowerment without transformation’, especially when unequal gender relations in farming communities are reproduced or remain unquestioned.

### 1.1 Conceptual and theoretical framework

This paper drew on Kabeer’s (1999) conceptualization of women’s empowerment and Connell’s concept of Hegemonic masculinity that provides for the theorization of masculinities (Connell and Messerschmidt, 2005). In her seminal work on *Resources, Agency, Achievements: Reflections on the Measurement of Women’s Empowerment*, Kabeer reflects on contestations around the concept women’s empowerment, especially the ways in which the concept’s feminist goals have been appropriated and applied within development discourse with mixed results. Citing limiting tendencies of conceptualizing empowerment as a ‘zero-sum’ game with politically weak winners and powerful losers or the instrumental grounds in which empowerment is seen as a means of achieving “broad set of desirable goals”, Kabeer set out to re-conceptualize what empowerment means and how it ought to be measured.

In her conceptual framework, she looks at empowerment along three indivisible domains – resources, agency, and achievements and how the concept itself is tied up with power. In effect, she looks at empowerment “in terms of the ability to make choices: to be disempowered, therefore, implies to be denied choice” (1999, p. 436). Consequently, Kabeer argues that “the notion of empowerment is that which is inescapably bound up with the condition of disempowerment and refers to the processes by which those who have been denied the ability to make choices acquire such an ability” (Kabeer, 1999, p. 437). In other words, empowerment entails a process of change these conceptual markers later informed how empowerment was adopted and applied in development practice, including agricultural research for development.

The second theoretical debate that informed the review is the concept of Masculinities (Connell, 1995; Connell and Messerschmidt, 2005). Connell (1995, 2005), pioneered the conceptualization of Hegemonic Masculinity as a model of understanding the social construction, hierarchy, and plurality of masculinities. Connell then defined hegemonic masculinity as a practice that legitimizes men’s dominant position in society and justifies the subordination of the common male population and women, and other marginalized groups of men. In this theory, Connell argues for the diversity, relationality and complexity of men’s behavior, practices, and identities (masculinities).

This approach has been embraced in agriculture research for instance by Saugeres (2003) and Cole et al. (2015). Saugeres notes that “the term masculinities refers to ideas and practices that enable some men to achieve and maintain a hegemonic position” (Saugeres, 2003, p. 145). The hegemonic model of masculinities acknowledges men as gendered and as products of socially changing environments, the hierarchy of masculinities (not one but multiple forms of masculinities) as well as entailing internal contradictions and the possibilities of movement toward gender democracy. The possibility of many forms of masculinities—see for instance what Brandth (2019) terms “Agricultural Masculinities,” or Lecoutere and Wuyts notion of “cooperative” and “Flexible husbands” or Cole et al. (2015), “rural Masculinities”—alerts us to the opportunity to examine ways in which the work on women’s empowerment, especially the call to involve men, has engaged with these diverse masculine identities and the implications of this deliberate and critical approach. The utility of the above theoretical approaches is illustrated in the analytical framework below.

### 1.2 Analytical framework

**Figure 1:**
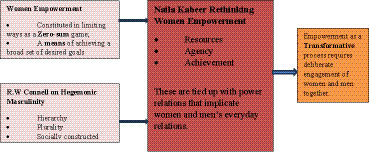
Analytic framework.

This paper critiques women’s epowerment approaches that constitute empowerment in limiting ways as a “zero-sum” game, as a means of achieving broader development goals or as something intelligible in relation to women only. Intervention that push for women’s empowerment without due attention to the everyday relations women have with men, end up polarizing women and men, cocealing the possibilities of knowing men’s social, hierarchical and plural experiences as gendered experiences (Connell and Messerschmidt, 2005). There are studies that indicate both men and women’s participation in paid and unpaid work though in varying proportions (Reddy et al., 2021). In her re-articulation of the notion of empowerment, Kabeer (1999) highlights three indivisible domains (Resources, Agency and Achievement) within which women and men’s everyday lives are intricately woven to the effect that for any meaningful empowerment to happen, it would require intentional focus on transforming norms and practices that underly not only women but also men’s behavior. This is particularly notable in Gender Transformative approaches’ call to engage men and women together (FAO et al., 2020) to facilitate critical reflections, learning and gender transformation (see Figure 1).

## 2 Research methodology

In order to select the papers for the review, the research team developed a guiding criterion that highlighted the categories of literature to search and engage with. Based on the key research questions, the research team marked out key aspects such as the search terms, content scope, geographical focus, inclusion criteria, and the databases from which to search literature for analysis.

### 2.1 Searching for literature

The team searched for appropriate literature using terms that included “Women’s empowerment in agriculture”, “masculinities and Agriculture”, “gender norms”, “agency” “Power relations”, “Rural masculinities” “male involvement in agriculture”. Literature search focused on four databases (AGORA, ScienceDirect, Google Scholar and Jstor) that popularly host agricultural research. The search also focused on content scope, deliberately looking out for literature on women empowerment in agriculture, masculinities, gender transformation, and nutrition-sensitive agriculture. In terms of geography, the study looked for literature on Sub Sahara Africa, South and South East Asia. In terms of scope, the analysis focused on literature published between 2010–2022, a period characterised by increased global calls on involving men in gender equality interventions to ensure transformation of social and gender norms (FAO et al., 2020).

The search yielded a total of 42,748 articles that were screened at multiple levels to determine those that would be included in the sample for review. Of the total articles above, 139 were selected for further screening, that is, scanning through the content to ensure the literature fits into the conceptual, geographical as well as the time span focused on in the study. Out of the 139 articles, 14 of them were finally selected for inclusion in the review sample. The 14 articles that were finally reviewed featured discussions on women’s empowerment and masculinities in agriculture. Some of the studies reviewed considered the concepts women empowerment and masculinities in isolation, while others explained how concepts are linked and applied in interconnected ways.

### 2.2 A synopsis of the literature reviewed

The literature reviewed can be categorized in three parts. The first set of literature includes authors that reflect on women’s empowerment in exclusion of men and masculinities. These include studies on food, nutrition, women’s reproductive capacities, and childcare in small holder agricultural households. See, for instance, Santoso et al. (2019), Sharaunga et al. (2015), Sraboni et al. (2014), and Van den Bold et al. (2015). Women’s empowerment is closely related to women’s improved abilities to make choices within the traditional feminine gender division of labor, e.g., food security, nutrition, child care, and less in questioning the root causes of unequal gender division of labor. There is notable silence around men’s everyday experiences in farming communities. The second set of literature includes studies that focus on the social gender norms that constrain women’s empowerment (Galiè et al., 2017; Hillenbrand and Miruka, 2019; Bonatti et al., 2019; Jeckoniah et al., 2012). Because of their dominant focus on gender and social norms in Agriculture, most of them reflect on forms of masculinities, femininities and patriarchal norms and practices that constrain women’s empowerment. In some cases, men are projected as obstacles and huddles to women’s empowerment with some interventions remaining blind to possibilities of transforming the unfair division of roles and spaces between women and men.

The third category of literature includes authors that explicitly engage with women’s empowerment and masculinities in agriculture. Some of these include Ambler et al. (2021), Casey et al. (2016), Cole et al. (2015), FAO et al. (2020), Lecoutere and Wuyts (2021), and Saugeres (2003). These deliberately name men and masculinities as implicated in the complex gender relations within the agricultural communities. They highlight diversity of masculinities and call for women and men joint decision-making in the households to ensure the transformation of unequal gender relations. For instance, in their recent collection on gender transformative approaches, FAO et al. (2020, 61) observe that “although women’s economic empowerment programming has powerful benefits on its own, it can be made even more effective at advancing gender equality when men are deliberately engaged as allies”. On their part, Ambler et al. (2021) critique the international practice in the women’s empowerment agenda where women’s empowerment work separates women from men, exclusively focusing on “women’s issues” even when women’s lives are intricately linked with men’s lives. ‘Deliberate’ engagement with the question of men and masculinities is thus seen as a step to enhance women’s empowerment programmes towards sustainable gender transformation.

## 3 Study findings

In this section, we share common themes that emerged in our review regarding the conceptualisation and application of women’s empowerment and masculinities independently and in an inter-connected manner within selected Agricultural studies.

### 3.1 Conceptualization of women’s empowerment, masculinities

Most agricultural research studies reviewed draw strongly on Naila Kabeer’s concept of women’s empowerment, highlighting the concept’s central focus on choice, voice, agency, and access to resources, especially where these entitlements were previously denied. In an article, the *Role of Women’s Empowerment in Child Nutrition Outcomes*, Santoso et al. (2019) define women’s empowerment as “the expansion in women’s ability to make strategic life choices” (2019, p. 1138). Authors indicate that this conceptualisation has gained global attention because of the intrinsic value women’s empowerment is thought to have on other social aspects. The article, thus, sets out to interrogate women’s empowerment as a tool (an enabler) and an instrumental way of achieving other social outcomes in society, e.g., child nutrition, and maternal health, among others. On their part, Lecoutere and Wuyts (2021) draw on Kabeer’s conceptualization to investigate the impact of introducing participatory intra household decision-making in Ugandan agricultural households on multiple dimensions of women’s empowerment. They define empowerment as “*a process of change where people acquire the ability to make strategic life choices* and *transform those choices into desired action and outcomes* to lead the life one has reason to value” (2021, p. 883). They particularly noted that empowerment bears real and/or perceived potential to challenge existing unequal power relations, and that it requires inner transformation from unquestioned acceptance of inequality or injustice to critical consciousness where women can at least imagine the possibility of choosing differently. In effect, empowerment encompasses the ability to have wider options to choose from, implying “the possibility of alternatives, the ability to have chosen otherwise” (Kabeer, 1999, p. 437). There is a noticeable silence on men and masculinities in articulation of the processes of change, even when these include women making strategic life choices in household settings.

The above definitions resonate with Ambler et al. (2021) understanding reflected in *Facilitating women’s access to an economic empowerment initiative* in Eastern Uganda. Ambler et al. (2021, p. 137) also raise the same idea of empowerment as a ‘process’ by which “those who have been denied the ability to make strategic life choices acquire such an ability”. What stands out different, though, is how the authors are quick to constitute women’s empowerment as a ‘means’ to achieving economic development. They argue that “women’s empowerment has long been recognized as an important element of economic development… and in recent years has gained increasing attention from major global bodies and development foundations leading to the increasing implementation of programs designed to boost women’s empowerment” (Ambler et al., 2021, p. 137). While Abler and others cite Kabeer (1999) in this debate, they possibly take for granted her caution on possibilities of economic instrumentalism that often crops up in women’s empowerment initiatives. Kabeer and indeed other feminist economists like Andrea Cornwall warned that certain forms of economic empowerment bear hallmarks of instrumentalism, especially when the intrinsic feminist goals of empowerment are pushed to the margins of economic development discourse (Cornwall, 2018; Cornwall and Rivas, 2015; Kabeer, 1999).

Cornwall (2018) characterizes this instrumentalist approach as a paradox that has entangled women’s empowerment discourse in a neoliberal global development context. She argues that the instrumental case for “investing in women and girls” is notable through an ever-growing parade of corporate actors, including major transnational corporations and investment banks, donors, NGOs, and philanthrocapitalists who extol the contributions women and girls make to development. “As feminist concepts such as “agency” and “choice” have come to be put to the service of neoliberalism, the word “empowerment” has been eviscerated of controversial or challenging content” (Cornwall, 2018, p. 1). She terms this kind of empowerment as “empowerment lite”—“a version of empowerment pared of any confrontation with the embedded social and power relations that produce societal and material inequities” (Cornwall, 2018, p. 3). These critical reflections begin to shed light on versions of empowerment that are stripped of the potential for gender transformation. It is notable that while aspects of women’s empowerment such as decision-making, agricultural production, ownership and control over resources, and leadership involve closer interactions between women and men, the definition of the concept is centered on women and offers limited room to engage with the understanding of men’s behaviors and practices that influence pathways to women’s empowerment.

### 3.2 Empowerment and possible legitimation of women’s domestic care work

Some publications on women’s empowerment in agriculture focus on outcomes related to food security, improved nutrition, childcare, maternal health, and education. Often, food security and nutrition projects address individual outcomes, with most studies focusing on the nutritional status of *women and children* (Bonatti et al., 2019; Santoso et al., 2019). This focus on the nutritional status of women helps to identify possibilities for improving both their lives and their nutritional access. However, in food security projects, the domestic environment where the femininities and masculinities are expressed, along with their influence and interconnections with malnutrition, is little studied. There is an assumption that femininities are directly connected to food and nutrition and that these are “women’s issues” (Bonatti et al., 2019) which presumably have nothing to do with men. Such an assumption that buttresses women’s empowerment is likely to ignore a focus on men in programmes on food security and nutrition, in effect consolidating women’s role in food production and entrenching further their domestication—the domestic and unpaid care work that we seek to challenge in the first place. In effect women’s empowerment may remain blind to possibilities of *transforming* the unfair division of roles and spaces between women and men. Empowerment programmes of this nature are more simplistic, focusing on the top, while ignoring the complex everyday relations in which women’s and men’s lives are entangled.

### 3.3 Women’s empowerment and masculinities

Not many studies deliberately conceptualize the notion of masculinities and later on link it with women’s empowerment, despite an increasing call to focus on men in women’s empowerment work (FAO et al., 2020). In the latter study, FAO and others present a compendium of good practices on Gender Transformative Approaches for Food Security, improved nutrition, and sustainable agriculture. They particularly mark out ‘engaging with couples,’ ‘working with’ women and men smallholder farmers’ and “engaging with men, boys and norm holders to address discriminatory gendered practices”. This is intended to enable women and men to move away from traditional roles and norms and live their lives differently. Authors argue that without involving the broader community, change is much more difficult, adding that “challenging the traditional views of masculinity enables men to live positively, and work and live with women as equals” (FAO et al., 2020, p. 15). These calls on gender transformative approaches to work with both men and women are repeated and emphasized as deliberate strategies to negotiate previous approaches that targeted women and men as binaries thus falling short on questioning and sustainably transforming deep-seated traditional masculine norms that define gender division of roles, entitlements and broader gender relations.

It is noticeable, however, that this hands-on guide for transforming gender relations in agriculture is only prescriptive in as far as *how* men can be drawn upon is concerned, as agricultural development practitioners work towards addressing discriminatory gendered practices. For instance, the compendium neither defines the concept of masculinities nor elaborates at length how masculinities come about, differ in forms and the social-cultural fluidity that surrounds this concept.

### 3.4 Empowerment in relation to men

Both FAO et al. (2020) and Sraboni et al. (2014) hint on the notion of empowerment in relation to men, even when empowerment has historically been used almost exclusively in relation to women. FAO et al. (2020) note that “[ensuring] women and men are both empowered by GTAs [Gender Transformative Approaches] creates sustained transformation. Once they have experienced the benefits of gender equality, most do not want to revert to previous practices” (2020, p. 14). Using the Women’s Empowerment Agricultural Index (WEAI) to measure women’s empowerment in Bangladesh, Sraboni et al. (2014, p. 21) argued: “[this] analysis has shown that the areas in which men and women are disempowered are quite different, with the implication that, depending on local context, different programmes and policies will need to be put in place to empower women and men alike”. The idea that the concept of empowerment can be used in relation to men, remains rare in the literature we analyzed, despite its potential to create sustainable transformation.

Other studies (Bonatti et al., 2019; Brandth, 2019; Cole et al., 2015; Lecoutere and Wuyts, 2021) use the concept of masculinity and talk of how masculinities, in particular toxic forms of masculinities, affect interventions intended to promote women’s empowerment. Despite the explicit mention of the concept in these studies on women’s empowerment in agriculture, these studies hardly engage with the conceptual and theoretical roots of the concept of masculinities. The concept of masculinity or masculinities owes its theoretical roots to Connell’s theory of hegemonic masculinities (but has since advanced beyond this model), which highlights social constructedness, relationality, and hierarchies that define norms, practices and behaviors associated with being a man (Connell, 1995). Connell defines Hegemonic masculinities as “the masculinity that occupies the hegemonic position in a given pattern of gender relations” (Connell, 1995, p. 74).

### 3.5 Explicit focus on masculinities

The other interesting work on masculinities in agriculture comes from Bonatti et al. (2019) who write about *Masculinities and Femininities in Food Insecurity Situations in Tanzania.* The study highlights linkages between gender and agriculture, especially when it indicates that the “current food insecurity is a consequence of many factors, including biophysical and social vulnerabilities related to climate change, water scarcity, increasing food prices, long-standing governmental neglect of agriculture, and gender inequality” (Bonatti et al., 2019, p. 2). They too, conceptualize gender (inequality) as a social cultural and structural issue (in effect moving it from an individual attribute to women) which entails “access to productive resources, income-generating and employment opportunities, time-use, and educational possibilities” (Bonatti et al., 2019, p. 2). It is notable how the authors acknowledge that while these relations have historically been analyzed focusing on women particularly in relation to the feminized role of food production, men too are implicated in access to productive resources, income generation, employment opportunities, and time use. Men’s stakes are reportedly higher when it comes to productive resources. Although women are the major food producers, and thus, significant contributors to food availability, men continue to play a dominant role in the decision-making process” (Bonatti et al., 2019, p. 2).

The above position acknowledges the gendered division of labour in which men are connected to the public world of work while women are connected to the private world, such as childcare, housekeeping, and nutritional needs of the household. This framing of gender relations has dominantly come to be known as women’s overwhelming participation in exploitative domestic care work and consequently informs international focus on how to address feminine disadvantage. Thus, Bonatti et al. critique the international practice in the women’s empowerment agenda where interventions separate women from men, exclusively focusing on what is perceived as “women’s issues” even when women’s lives are intricately linked with men’s lives. In this critique, Bonatti and colleagues demonstrate ways in which femininities and masculinities are interconnected subjectivities that are directly linked with food insecurity in the household. By picking up on this, the authors emphasize the need to pay attention to the interconnections amongst genders, especially in the women’s empowerment movement, to avoid *simplifying* complex social relations of inequality as just “women’s issues.” This is a strong case for a broader and inclusive approach to women’s empowerment which is reiterated by FAO et al. (2020).

On their part, Cole et al. (2015) explicitly interrogate the concept of masculinities in agricultural communities by tracing its conceptual roots in Connell’s hegemonic masculinities framework and demonstrating how masculinities are socially constructed in relation to women’s lives and experiences. They explore an intricate relationship between poverty, gender inequality, and rural masculinities in aquatic agricultural systems in Zambia. Their work uses the concept of masculinity in relation to Connell’s (1995) “hegemonic” masculinities as it has earlier been defined. The authors also talked of the concept of ‘masculine rural’ to help highlight one way masculinity is being constructed in rural study settings and its implications for women’s lived experiences. They argue that “often, the masculine in the rural is associated with the value placed on “hard physical labour, toughness, tenacity, dependability, strength, and the need to conquer or overcome nature and exert control over the machines or equipment that make this possible, as well as the ability to succeed as a farmer” (Cole et al., 2015, p. 158). Importantly, this study helps us appreciate the changing nature of masculinities within agriculture. It demonstrates this using the notion of being a ‘big man’ in a rural, southern African setting—a person who is powerful, chief-like, demands respect, is married (perhaps to multiple women) and head of a household, accumulates wealth through people (e.g., children, spouse), and owns or controls assets such as land, cattle, and farming equipment. They note that “[In] changing economic and natural resource contexts where farming provides fewer viable occupational choices and women are increasingly taking up household and farming responsibilities for a number of reasons, some men are experiencing identity discontinuities” (Cole et al., 2015, p. 158). These illustrations point to ways in which men are implicated in the conceptualisation of gender and in the changing gender relations to the effect that when men’s lives change, these changes affect their identity, their sense of being, and belonging, as much as they do to women.

Studies by Bonatti et al. (2019) and Cole et al. (2015) demonstrate explicit links between men’s and women’s lives. These intricate relations have implications for agricultural programming. For instance, focusing on women’s lives through empowerment programmes without deliberate consideration of norms and practices that regulate men’s behaviors and relations with each other and with women has the potential to constrain meaningful transformation of gender relations.

## 4 Discussion of findings

### 4.1 Empowerment without transformation?

This study set out to interrogate existing literature on women empowerment and masculinities and examine possibilities of empowerment initiatives that fall short of transforming gender power relations. We have noted that despite the milestones in the application of women empowerment in ways that engage both women and men, it is notable that some of the authors’ analysis and study conclusions are limited only to women, consequently constituting ‘men’ in these ‘participatory’ and ‘inclusive’ approaches as a tool or means to facilitate women’s empowerment. There is also a notable pattern in which women’s empowerment is conceptualized based on the traditional gender division of roles. Often, this approach looks at women’s empowerment drawing on existing societal division of labour between women and men in which women are seen as the ordained childcare providers for example. Other gender based agricultural initiatives include activities that target women and nutrition, women small holder farmers for subsistance agriculture, women in informal agriculture, women’s crops, women plots, among others. By taking this approach, men and men’s roles are not seen as central in the empowerment process. Indeed, Santoso et al. (2019, p. 1141) acknowledge that “[currently], the evidence seems to indicate that women, when given the opportunity, make better decisions *than* [*our emphasis*] men about the care and nutrition of children”. Authors suggest, this is due to the assignment of child care as a woman’s task in many communities in the world. That women are the ones who need empowerment to increase their life choices and capacities to actively participate in certain social roles for example, childcare, allerts us to potential essentialisms where women are constituted as child nurturers and limits the potential to transform unequal allocation of roles that have been socially constituted as ‘women’s roles’. It also limits the possibility of transforming gender roles in agricultural households.

There is a common pattern in which the notion of “male involvement”/ masculinities is used in agricultural research just in passing, perhaps as a buzzword with no detailed explanation of *what* it is, the theory behind it, why, and the kind of transformation. For some studies, as earlier indicated, the concept of masculinities is not defined at all. Questions such as who is a man, in which context? How are men’s behaviors and practices constructed, nurtured, and sustained? What is the impact of different forms of men’s behaviors and practices on men themselves, those they interact and relate with and on development processes? How do we conceptualize men’s empowerment? are rarely engaged with. Yet, these omissions are not without effect. They entrench the idea of involving men in gender equality and women’s empowerment in agriculture (and indeed in other sectors) as a cosmetic and rhetorical call, one that has no potential to challenge deep-rooted social and gender norms that regulate men and women’s behaviors and practices. Both inadequate questioning and inattention to masculinities in women empowerment work have the potential to reproduce male domination or promote forms of empowerment unable to subvert rooted male hegemony such as men’s disproportionate ownership and control over agricultural resources, unfair inheritance regimes, women’s limited participation in decisions around productive agricultural resources. In societies where seclusion of women is the norm, women are dependent on a family middleman for all communication external to the household, including accessing agricultural information on farming practices, loans and markets.

## 5 Conclusion

The literature we reviewed shows a growing body of knowledge on how women’s empowerment, masculinities, and gender transformative approaches have inspired conceptualisation of agricultural research and development practice. Nonetheless, these concepts remain largely unexamined in particular their conceptual interconnectivity, indivisibility, and contextuality and their rootedness in culture. It is important that research and interventions on women’s empowerment endeavors to put on a masculinities lens that helps explain the complex relations between women and men’s lives in agricultural communities. Unpacking each of these concepts, tracing their conceptual and theoretical roots, and appreciating their interconnectedness has the potential to inform evidence-based agricultural interventions to transform gender inequalities in agriculture.

## References

[ref1] Ambler K. Jones K. O’Sullivan M. (2021). Facilitating women’s access to an economic empowerment initiative: evidence from Uganda. World Dev. 138, 1–13. doi: 10.1016/j.worlddev.2020.105224

[ref2] Bonatti M. Borba J. Schlindwein I. Rybak C. Sieber S. (2019). “They came home over-empowered”: identifying masculinities and femininities in food insecurity situations in Tanzania. Sustainability 11, 1–15. doi: 10.3390/su11154196

[ref4] Brandth B. (2019). Tough and tender’? Agricultural masculinities and fathering activities. Int. J. Mascul. Stud. 14, 223–238. doi: 10.1080/18902138.2019.1654725

[ref5] Casey E. Carlson J. Bulls S. T. Yager A. (2016). Gender transformative approaches to engaging men in gender-based violence prevention: a review and conceptual model. Trauma Violence Abuse 19, 231–246. doi: 10.1177/1524838016650191, 27197533

[ref8] Cole S. Puskur R. Rajaratnam S. Zulu F. (2015). Exploring the intricate relationship between poverty, gender inequality and rural masculinity: a case study from an aquatic agricultural system in Zambia. Cult. Soc. Masc. 7, 154–170.

[ref9] Connell R. (1995). Masculinities. 1st Edn. Cambridge, UK: Polity Press.

[ref001] Connell R. W. (2005). Masculinities, (2nd Edition), Cambridge, UK: Polity Press.

[ref10] Connell R. W. Messerschmidt J. W. (2005). Hegemonic masculinity: rethinking the concept. Gender Soc. 19, 829–859. doi: 10.1177/0891243205278639

[ref11] Cornwall A. (2000). Missing men? Reflections on men, masculinities and gender in GAD. IDS Bull. 31, 18–27. doi: 10.1111/j.1759-5436.2000.mp31002003.x

[ref12] Cornwall A. (2018). Beyond “empowerment lite”: women’s empowerment, neoliberal development and global justice. Cad. Pagu 52, 1–30. doi: 10.1590/18094449201800520002

[ref13] Cornwall A. Rivas A. (2015). From ‘gender equality and women’s empowerment’ to global justice: reclaiming a transformative agenda for gender and development. Third World Q. 36, 396–415. doi: 10.1080/01436597.2015.1013341

[ref15] FAO, IFAD, & WFP. (2020). Gender transformative approaches for food security, improved nutrition and sustainable agriculture – A compendium of fifteen good practices. Rome, Italy: FAO, IFAD, WFP.

[ref16] Galiè A. Jiggins J. Struik P. C. Grando S. Ceccarelli S. (2017). Women’s empowerment through seed improvement and seed governance: evidence from participatory barley breeding in pre-war Syria. NJAS Wageningen J. Life Sci. 81, 1–8. doi: 10.1016/j.njas.2017.01.002

[ref17] Hillenbrand E. Miruka M. (2019). “Gender and social norms in agriculture: a review” in 2019 annual trends and outlook report: Gender equality in rural Africa: From commitments to outcomes. eds. Quisumbing A. R. Meinzen-Dick R. S. Njuki J. (Washington, DC: International Food Policy Research Institute (IFPRI)), 11–31.

[ref18] Jeckoniah J. N. Nombo C. I. Mdoe N. S. Y. (2012). Women empowerment in agricultural value chains: voices from onion growers in northern Tanzania. Res. Humanit. Soc. Sci. 2, 54–59.

[ref19] Kabeer N. (1999). Resources, agency, achievements: reflections on the measurement of women’s empowerment. Dev. Change 30, 435–464. doi: 10.1111/1467-7660.00125

[ref21] Lecoutere E. Wuyts E. (2021). Confronting the wall of patriarchy: does participatory intrahousehold decision making empower women in agricultural households? J. Dev. Stud. 57, 882–905. doi: 10.1080/00220388.2020.1849620

[ref23] Reddy A. A. Mittal S. Singha Roy N. Kanjilal-Bhaduri S. (2021). Time allocation between paid and unpaid work among men and women: an empirical study of Indian villages. Sustainability 13:2671. doi: 10.3390/su13052671

[ref24] Santoso M. V. Kerr R. B. Hoddinott J. Garigipati P. Olmos S. Young S. L. (2019). Role of women’s empowerment in child nutrition outcomes: a systematic review. Adv. Nutr. 10, 1138–1151. doi: 10.1093/advances/nmz056, 31298299 PMC6855975

[ref25] Saugeres L. (2003). Of tractors and men: masculinity, technology and power in a French farming community. Sociol. Ruralis 42, 143–159. doi: 10.1111/1467-9523.00207

[ref26] Sharaunga S. Mudhara M. Bogale A. (2015). The impact of ‘women’s empowerment in agriculture’ on household vulnerability to food insecurity in the KwaZulu-Natal Province. Forum Dev. Stud. 42, 195–223. doi: 10.1080/08039410.2014.997792

[ref27] Sraboni E. Quisumbing A. R. Ahmed A. U. (2014). How empowered are Bangladeshi women in the agricultural setting? Empirical evidence using a new index. Bangladesh Dev. Sud. 37, 1–25.

[ref28] Van den Bold M. Dillon A. Olney D. Ouedraogo M. Pedehombga A. Quisumbing A. (2015). Can integrated agriculture-nutrition programmes change gender norms on land and asset ownership? Evidence from Burkina Faso. J. Dev. Stud. 51, 1155–1174. doi: 10.1080/00220388.2015.1036036, 30363952 PMC6183935

