# Supplementary material for: “Empowerment” without transformation? A critique of women empowerment without a masculinities lens in gender and agriculture literature
Source: Front Sociol. 2026 Feb 20;11:1636863. doi: 10.3389/fsoc.2026.1636863 (PMC12962902; doi:10.3389/fsoc.2026.1636863)
Supplement: Supplementary file 1 [file Supplementary_file_1.pdf]

Appendix 1: List of studies reviewed

Studies analysed during literature review

1. Ambler, K., Jones, K., & O'Sullivan, M. (2021). Facilitating women's access to an economic empowerment initiative: Evidence from Uganda. *World Development*, *138*. https://doi.org/10.1016/j.worlddev.2020.105224
2. Bold, M. V. A. N. D. E. N., Dillon, A., Olney, D., Ouedraogo, M., Pedehombga, A., & Quisumbing, A. (2015). Can Integrated Agriculture-Nutrition Programmes Change Gender Norms on Land and Asset Ownership ? Evidence from Burkina Faso. *The Journal of Development Studies*, *51*(9), 1155–1174.
3. Bonatti, M., Borba, J., Schlindwein, I., Rybak, C., & Sieber, S. (2019). "They came home over-empowered": Identifying masculinities and femininities in food insecurity situations in Tanzania. *Sustainability*, *11*(15). https://doi.org/10.3390/su11154196
4. Casey, E., Carlson, J., Bulls, S. T., & Yager, A. (2016). *Gender Transformative Approaches to Engaging Men in Gender-Based Violence Prevention : A Review and Conceptual Model*. https://doi.org/10.1177/1524838016650191
5. Cole, S., Puskur, R., Rajaratnam, S., & Zulu, F. (2015). Exploring the intricate relationship between poverty, gender inequality and rural masculinity: a case study from an aquatic agricultural system in Zambia. *Culture, Society & Masculinities*, *7*(2), 154–170.
6. FAO, IFAD, & WFP. (2020). *Gender transformative approaches for food security, improved nutrition and sustainable agriculture – A compendium of fifteen good practices.* https://doi.org/10.4060/cb1331en
7. Galiè, A., Jiggins, J., Struik, P. C., Grando, S., & Ceccarelli, S. (2017). "Women's empowerment through seed improvement and seed governance: Evidence from participatory barley breeding in pre-war Syria." *NJAS - Wageningen Journal of Life Sciences*, *81*, 1–8. https://doi.org/10.1016/j.njas.2017.01.002
8. Hillenbrand, E., & Miruka, M. (2019). Gender and social norms in Agriculture: A review. In A. R. Quisumbing, Meinzen-Dick, R. Suseela, & J. Njuki (Eds.), *2019 Annual trends and outlook report: Gender equality in rural Africa: From commitments to outcomes* (pp. 11–31). International Food Policy Research Institute (IFPRI). https://ebrary.ifpri.org/utils/getfile/collection/p15738coll2/id/133472/filename/133680.pdf
9. Jeckoniah, J. N., Nombo, C. I., & Mdoe, N. S. Y. (2012). Women Empowerment in Agricultural Value Chains: Voices from Onion Growers in Northern Tanzania. *Research on Humanities and Social Sciences*, *2*, 54–59.
10. Lecoutere, E., & Wuyts, E. (2021). Confronting the Wall of Patriarchy: Does Participatory Intrahousehold Decision Making Empower Women in Agricultural Households? *Journal of Development Studies*, *57*(6), 882–905. https://doi.org/10.1080/00220388.2020.1849620
11. Santoso, M. V., Kerr, R. B., Hoddinott, J., Garigipati, P., Olmos, S., & Young, S. L. (2019). Role of Women's Empowerment in Child Nutrition Outcomes: A Systematic Review. *Advances in Nutrition*, *10*(6), 1138–1151. https://doi.org/10.1093/advances/nmz056
12. Saugeres, L. (2003). Of tractors and men: Masculinity, technology and power in a French farming community. *Sociologia Ruralis*, *42*(2), 143–159. https://doi.org/10.1111/1467-9523.00207
13. Sharaunga, S., Mudhara, M., & Bogale, A. (2015). The Impact of 'Women's Empowerment in Agriculture' on Household Vulnerability to Food Insecurity in the KwaZulu-Natal Province,. *Forum for Development Studies*, *42*(2), 195–223.
14. Sraboni, E., Quisumbing, A. R., & Ahmed, A. U. (2014). How Empowered are Bangladeshi Women in the Agricultural Setting ? Empirical Evidence using a New Index. *The Bangladesh Development Sudies*, *37*(3), 1–25.
